# Long-term impact of battle injuries; Ten-year follow-up of Dutch servicemembers injured in Afghanistan

**DOI:** 10.1371/journal.pone.0334622

**Published:** 2025-11-12

**Authors:** Benjamin L. Turner, Thijs T. C. F. van Dongen, Floris Idenburg, Loes de Kruijff, Eelco P. Huizinga, Eric Vermetten, Erik Hoencamp, Rigo Hoencamp

**Affiliations:** 1 Diving Medical Centre, Royal Netherlands Navy, Den Helder, the Netherlands; 2 Defence Healthcare Organisation, Ministry of Defence, Utrecht, the Netherlands; 3 Department of Surgery, Leiden University Medical Centre, Leiden, the Netherlands; 4 Rehabilitation Centre de Hoogstraat, Utrecht, the Netherlands; 5 Department of Surgery, Central Military Hospital, Ministry of Defence, Utrecht, the Netherlands; 6 Department of Psychiatry, Leiden University Medical Centre, Leiden, the Netherlands; 7 Institute of Psychology, Leiden University, Leiden, The Netherlands; 8 Department of Surgery, Alrijne Hospital, Leiderdorp, the Netherlands; 9 Trauma Research Unit, Department of Surgery, Erasmus MC, University Medical Centre Rotterdam, Rotterdam, the Netherlands; Uniformed Services University: Uniformed Services University of the Health Sciences, UNITED STATES OF AMERICA

## Abstract

**Background:**

Our five-year follow-up of injured service members deployed to Afghanistan showed that battlefield casualties (BCs) experience lower quality of life and higher distress than non-injured peers. This ten-year follow-up aims to extend the understanding of the long-term impact of battlefield injuries and to enrich the knowledge of long-term impacts of combat wounds.

**Methods:**

Dutch service members deployed to Afghanistan (2006–2010) were divided into groups: injured by hostile actions (BCs) and two control groups—non-injured combat unit members (CG1) and non-injured staff (CG2). Data on injuries, demographics, and rank were gathered, and participants completed an online survey covering trauma, reintegration, distress, quality of life and physical and mental health. Group differences were assessed using Kruskall-Wallis tests and ANOVA.

**Results:**

The mean age of participants in the BC group (n = 47) was lower than CG1 (n = 95) and CG2 (n = 64). BCs reported significantly higher levels of distress compared to control groups. Of 25 significantly different scores between the three subgroups in our five-year follow-up, all but the Work Positive PDRS subscore remained significantly different compared with the control group. Additionally, the EQ-6D and General Health SF-36 subscore were significant at five years but not this study.

**Conclusion:**

Service members with battlefield injuries report significantly lower quality of life, predominantly in physical functioning, and higher psychological distress compared to non-injured peers. Most differences between BCs and non-injured peers identified in our 5Y follow-up remained significantly different. We provide recommendations to improve the quality of aftercare based on our observations (1) periodical evaluation every 3–5 years, (2) comprehensive assessment of care needs and (3) balancing disease burden and health to optimize societal and workplace integration.

## Background

Military medicine is a multi-dimensional field, ranging from acute trauma care to long-term physical and mental rehabilitation. The care for military personnel injured during deployments in conflict areas, referred to as battlefield casualties (BCs), therefore does not end on the battlefield or at the Role 3 Medical Treatment Facility (MTF). On the contrary, military medical professionals are also responsible for long-term follow-up and parts of post-injury reintegration into service and/or society [1].

After the full-scale armed conflict in Afghanistan ended, the chance of large-scale military conflicts involving NATO forces was considered to be low by many. However, developments in Ukraine and rising tensions across the globe such as in and around the Levant, show that kinetic warfare is far from over. The impact of such conflicts on society, including battlefield casualties, in the short- and long term becomes all the more relevant.

In 2015, our battlefield casualty research group determined a lack of studies offering systematic comparison of long-term follow-up of injured service members as well as a lack in comparing BCs to a comparable group of non-injured service members from the same operational theatre. To assess the quality of long-term military medical and psychological care for BCs and to identify areas of improvement for quality of care, a five-year follow-up study was conducted. Assessing the long-term impact of events showed the BC group reported a significantly lower QOL and significantly higher distress levels [2].

Conducting an extended observation period will enable us to gain a more comprehensive understanding of the long-term impact of battlefield injuries and allow us to assess longer-term outcomes and progression of the effects of battlefield injuries over time. Additionally, the aging of the study population, and therefore the increased time between the moment of injury and the follow-up may reveal new findings. The insights gained from this ten-year follow-up will potentially guide us toward valuable new research avenues to enhance the quality of care for those who become injured while serving their country.

This study therefore aims to assess the ten-year follow-up of QOL of service members injured on the battlefield, compare it with the five-year follow-up of BCs as well as compare it to a group of non-injured peers.

## Methods

### Study design and participants

The population of this nested cross-sectional survey study consists of Dutch military service members who served between 2006–2010. During this period, The Netherlands deployed around 17,000 troops to Afghanistan as part of Task Force Uruzgan (TFU) [3]. The participants in this study and in the 5-year follow-up do not fully overlap, with some individuals participating in only one of the two assessments.

The general digital admission database of the Dutch Ministry of Defence (MOD) was used to identify potential participants, filtering for battlefield casualties between August 2006 and August 2010. To specify the sustained injuries data were collected on mechanism of injury (MOI), anatomical distribution of wounds (AD), and Injury Severity Score (ISS).

An independent employee from the department of epidemiology of the MOD randomly selected the control groups, excluding service members who sustained (a battlefield) injury.

All eligible participants were asked to provide written informed consent and complete an online questionnaire between the fourth quarter of 2021 to the third quarter of 2022, receiving two digital reminders if necessary. Sex, age, marital status, and educational level were measured, and the participants were divided into three rank groups, corresponding to their rank during deployment: junior enlisted (E1-E4), senior enlisted and non-commissioned officers (E5-WO2) and officers (O1-O10).

### Inclusion and exclusion criteria

All included service members were deployed to a combat zone and were distributed over three groups in this study (1) service members that were injured in theatre as a result of hostile action, labelled as BCs, (2) non-injured service members, from combat units (control group CG1) and (3) non-injured service members, with a staff function (control group CG2). The exclusion criterium was having sustained a disease or non-battlefield injury (DNBI).

A service member was included in the BC group if they sustained a battlefield injury, being defined as an injury because of hostile action by enemy forces. This hostile action could have been sustained either in combat, going to combat or returning from a combat mission, for example by an improvised explosive device (IED). Servicemembers in CG1 or CG2 may have served in the same units as those in the BC group but were not injured themselves.

### Assessment

The self-reported survey contained four domains: (1) the Impact of Event Scale (IES) [4], (2) the Post Deployment Reintegration Scale (PDRS) [5,6], (3) the Symptom Checklist 90 (SCL-90) [7–9], (4) Quality of Life measured using the EuroQol-6D (EQ-6D)^9^ and the 36-item Short Form health Survey (SF-36) [10].

The IES [4] is a 22-item measurement tool that assesses impact of traumatic stress. Participants answer on a 5-point scale, scoring 0 (not at all) to 4 (extreme), which renders a total score (zero to 88). This total score is subdivided into the subscales intrusion (INT), avoidance (AVO) and hyper arousal (HAR).

The PDRS [5], consisting of 36 items, is a multidimensional assessment-tool of post deployment reintegration attitude that reflects the experience of military personnel in multiple domains (Work negative [WN]; Work positive [WP]; Family negative [FN]; Family positive [FP]; Personal negative [PN], and Personal positive [PP]). Each domain is divided into positive and negative subscales (scored 0–5). A higher score on the negative subscale indicates a more negative attitude, and higher scores on positive subscales indicate more positive attitudes [5].

The SCL-90 [7–9] contains 90 questions with a 5-point rating scale (1 (not at all) to 5 (extreme)). It is used to assess both physical and psychological symptoms of distress. The outcome scores are divided into nine symptom subscales: anxiety (ANX, range 10–50), agoraphobia (AGO, range 7–35), depression (DEP, range 16–80), somatization (SOM, range 12–60), insufficient thinking and handling (IN, range 9–45), distrust and interpersonal sensitivity (SEN, range 18–90), hostility (HOS, range 6–30), sleeping disorders (SLE, range 3–15), and a rest subscale (REST, range 9–45). Total score (SCL-90-TOT) ranges 90–450.

The entire EQ-6D questionnaire was utilized, including a visual analogue scale evaluating health-related quality of life and health preferences. The SF-36 measures the health status of the person, and is often used to calculate the cost-effectiveness of treatment [11]. Eight scaled scores between 0–100, 0 being maximum disability and 100 being no disability, are derived from the weighted sums of the questions in the survey sections. The sections are: vitality, physical functioning, bodily pain, general health, physical role functioning, emotional role functioning, social role functioning, and mental health.

### Statistical analysis

Data were accessed for research purposes between 04/07/2024 and 01/02/2025. Demographics are described in Table 1. Variables with a continuous distribution were summarized using mean and standard deviation (SD). Nominal and ordinal variables were described using absolute and relative frequencies.

**Table 1:** Demographics of battle casualties and both control group.

Characteristic during deployment	BC N = 47	CG1 N = 95	CG2 N = 64	P-value
**Age**, mean (range)	36.5 (30 - 61)	40.2 (30–65)	48.2 (31- 70)	< 0.05
**Sex (%)**				> 0.05
Male	46 (97.9%)	89 (93.7%)	59 (92.2%)	
Female	1 (2.1%)	3 (3.2%)	4 (6.3%)	
Other	0	3 (3.2%)	1 (1.6%)	
**Marital Status (%)**				> 0.05
Married/Registered partner	30 (63.8%)	68 (71.6%)	50 (78.1%)	
Relationship	10 (21.3%)	16 (16.8%)	7 (10.9%)	
Single	6 (12.8%)	7 (7.4%)	6 (9.4%)	
Divorced	1 (2.1%)	4 (4.2%)	1 (1.6%)	
Widow	0	0	0	
**Active duty (%)**	14 (29.8%)	49 (51.6%)	22 (34.4%)	< 0.05
**Rank (%)**				< 0.05
Junior enlisted	17 (36.2%)	26 (27.4%)	5 (7.8%)	
Senior enlisted and Non-commissioned officers	27 (57.4%)	49 (51.6%)	41 (64.1%)	
Officers	3 (6.4%)	20 (21.1%)	18 (28.1%)	
**Number of deployments,** mean (range)	2.8 (1 –8)	3.4 (1 –13 )	3.4 (1 –13)	> 0.05
**Educational status (%)**				< 0.05
Low	8 (17.0%)	3 (3.2%)	28 (43.8%)	
Middle	26 (55.3%)	67 (70.5%)	10 (15.6%)	
High	13 (27.7%)	25 (26.3%)	26 (40.6%)	

No power analysis was performed: the survey was distributed to the largest possible group within the target population and all responses were included in the study.

Statistical analyses were performed using SPSS (version 25, IBM Corporation, Armonk, New York) and R (version 4.4.2, R Core Team). Assessment of differences in baseline characteristics of the groups were determined using the chi-square test for sex, active-duty status, rank and educational status. Fisher’s exact test was used for marital status due to a low number of divorced and widowed participants. The variables for age and number of deployments were not normally distributed and were analysed using the Kruskal-Wallis test.

The assessment of differences in the outcomes between the BC group and both control groups was performed using the Kruskal Wallis test, due to their non-normal distribution.

The methodology employed in our statistical analysis was reviewed and validated by an external statistician.

### Ethics approval

This study was approved by the Dutch Ministry of Defence (MOD) and the Institutional Review Board and the Medical Ethics Committee of Leiden University, the Netherlands under p11.184.

## Results

**Fig 1:**
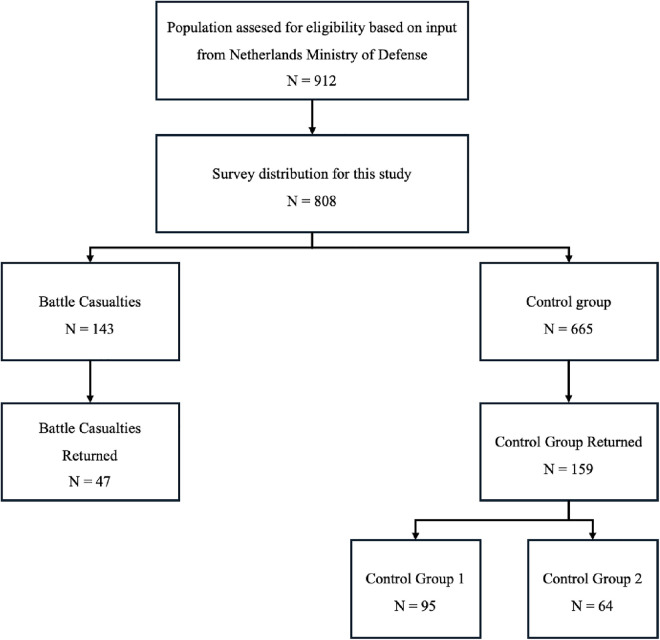
Survey distribution.

All surveys were electronically distributed, of which 143 were sent to the BC group (47 responses = 32.8%) and 665 to potential CG1/CG2 respondents (159 responses = 23.9%) (Fig 1). The number of potential participants was smaller than in our 5Y follow-up due to (1) administrative inconsistencies within the general admission database, resulting in an inability to contact certain participants or (2) mortality. A maximum of 2 reminders was sent to each participant who did not (initially) respond. The CG participants were significantly older than the BC participants. More males than females were represented in each group. 29.8% of BCs were still on active duty, compared to 51.6% of CG1 and 34.4% of CG2. Significant differences between the groups were observed in age, rank, current active-duty status, and educational level. Non-commissioned officers (NCO) represented the largest demographic subset in all groups. The average number of deployments for each participant was 3, ranging from 1 to 13.

The most frequent mechanisms of injury, with multiple mechanisms being possible per injury, were blast (43/47), fragments (9/47) and small arms fire (3/47).

**Fig 2:**
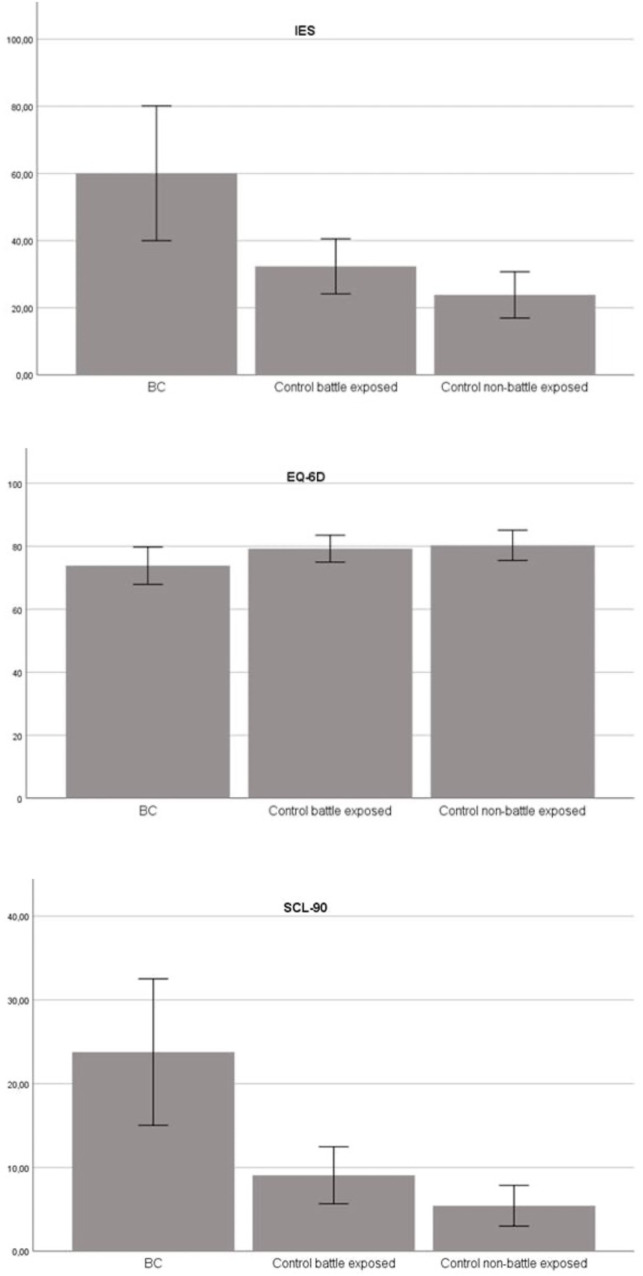
Schematic overview of mean scores of the IES (Impact of Event Scale), SCL-90 (Symptom Checklist-90), and EQ-6D (EuroQol 6-Dimension) per subgroup.

The mean scores for the IES, SCL90 and EQ-6D questionnaires are schematically depicted in Fig 2. Significant differences (p < 0.05) between the three groups were shown for the mean IES scores, respectively 23.7 (SD 28.8) in the BC group, 9.1 (SD 15.6) for CG1, and 5.4 (SD 9.5) for CG2. All IES subscales differed significantly between the groups, as well as for all negative PDRS subscores and the Work Positive subscore (Table 2).

**Table 2:** Scores of IES, PDRS, SCL-90, EQ-6D per subgroup.

Variable Mean (SD)	BC N = 47	CG1 N = 95	CG2 N = 64	P value	Significant difference in 5Y follow-up
IES	23.773 (28.761)	9.060 (15.586)	5.426 (9.491)	0.001Φ	Yes
*INT*	0.886 (1.121)	0.384 (0.591)	0.227 (0.446)	0.001Φ	Yes
*AVO*	0.901 (1.153)	0.371 (0.707)	0.158 (0.354)	0.000Φ	Yes
*HAR*	0.616 (0.789)	0.209 (0.476)	0.127 (0.306)	0.000Φ	Yes
PDRS					
*WP*	3.732 (0.582)	3.669 (0.719)	3.378 (0.742)	0.014Φ*	No
*WN*	2.830 (0.970)	2.507 (0.896)	2.164 (0.879)	0.001Φ	Yes
*FP*	3.120 (0.810)	3.088 (0.906)	2.831 (0.854)	0.215	No
*FN*	2.649 (1.041)	2.081 (0.817)	1.796 (0.851)	0.000Φ	Yes
*PP*	3.366 (0.838)	3.486 (0.836)	3.452 (0.830)	0.783	No
*PN*	2.677 (1.152)	2.176 (0.943)	1.937 (0.870)	0.002Φ	Yes
SCL-90	60.071 (64.395)	32.358 (37.047)	23.830 (26.475)	0.002Φ	Yes
*ANX*	5.905 (7.867)	2.926 (4.485)	1.729 (2.778)	0.003Φ	Yes
*AGO*	3.167 (5.780)	1.271 (3.435)	0.491 (1.265)	0.001Φ	Yes
*DEP*	10.881 (13.151)	5.741 (8.393)	4.661 (6.725)	0.031Φ	Yes
*SOM*	8.809 (9.405)	4.630 (5.163)	3.966 (4.210)	0.035Φ	Yes
*IN*	8.452 (7.816)	4.716 (5.650)	3.898 (4.432)	0.002Φ	Yes
*SEN*	10.071 (12.066)	6.716 (7.678)	4.712 (5.318)	0.015Φ	Yes
*HOS*	4.333 (5.088)	1.877 (3.014)	1.373 (2.059)	0.000Φ	Yes
*SLE*	3.738 (3.500)	2.136 (2.845)	1.559 (2.284)	0.002Φ	Yes
*REST*	4.714 (5.320)	2.346 (3.351)	1.441 (1.878)	0.002Φ	Yes
EQ-6D	73.84 (19.739)	79.22 (19.786)	80.29 (18.895)	0.067*	Yes
SF-36					
*PF*	80.89 (23.700)	96.24 (7.319)	93.230 (12.351)	0.000Φ	Yes
*SF*	75.556 (26.909)	86.938 (21.807)	88.911 (20.258)	0.002Φ	Yes
*RP*	63.33 (44.467)	90.170 (26.279)	80.650 (34.894)	0.000Φ	Yes
*RE*	65.926 (46.324)	88.390 (28.912)	87.634 (29.093)	0.003Φ	Yes
*MH*	68.98 (22.838)	81.570 (15.984)	82.060 (15.360)	0.001Φ	Yes
*VT*	58.56 (22.729)	70.900 (19.880)	74.350 (19.216)	0.000Φ	Yes
*BP*	70.023 (27.300)	87.274 (16.600)	83.706 (20.028)	0.001Φ	Yes
*GH*	64.89 (21.807)	72.020 (20.612)	72.820 (19.807)	0.111*	Yes

Φ indicates significance, * indicates significance in 10Y follow-up without significance in 5Y follow-up or vice verca, BC indicates battle casualty, CG1: control group 1, CG2: control group 2, SD: standard deviation, N = number, *Subscales IES, INT: intrusion; AVO: avoidance, HAR: hyper arousal.

**Subscales PDRS WP: Work positive, WN: Work negative, FP: Family positive, FN: Family negative, PP: Personal positive, PN: Personal negative. ***Subscales SCL-90 ANX: anxiety, AGO: agoraphobia, DEP: depression, SOM: somatization, IN: insufficient thinking and handling, SEN: distrust and interpersonal sensitivity, HOS: hostility. SLE: sleeping disorders REST: rest subscale. ****Subscales SF-36 PF: Physical functioning SF: Social functioning, RP: Role physical, RE: Role emotional, MH: Mental health VT: Vitality, BP: Bodily pain, GH: General Health.

The mean SCL-90 score for the BC group, 601 (SD 64.4), was significantly higher than the mean scores of CG1 (32.4, SD: 37.1) and CG2 (23.8, SD:26.5). All SCL-90 subscores were significantly higher in the BC group compared to CG1 and CG2.

The mean quality of life score represented in the EQ6-D was lower in the BC group (74.4 SD: 19.7) but not significantly different compared to CG1 (79.2 SD: 19.8) and CG2 (80.3 SD:18.9) (p = 0.067). Finally, all SF-36 subscales, except for General Health were significantly lower in the BC group compared to CG1 and CG2.

### Key differences between 5-year and 10-year follow-up

Table 2 depicts whether the subscale score differences were shown to be significantly different among the subgroups in our 5Y follow-up. In this 10-year follow-up, we identify a total of 25 statistically significant scores between the groups. Of these, all except the Work Positive PDRS subscore were identified in our 5Y follow-up. Additionally, two measures, the EQ-6D and the GH subscore of the SF-36, were found to be significant in the 5Y follow-up but were not revealed in this study.

## Discussion

In this study a 10-year follow-up analysis of a group of Dutch service members injured in combat. The results of an earlier 5-year follow-up study were available and published in 2014, providing us with the preplanned unique opportunity to compare outcome and the impact of battlefield injuries over time in order the provide long term care for our (injured) service members.

The BC group reported significantly higher traumatic stress levels measured by IES and SCL90 compared to both control groups. Furthermore, all negative PDRS subscales showed significant differences between subgroups, indicating that injured servicemembers experience more negative impact from their battlefield experiences than their non-injured peers. The overall quality of life in the BC group was significantly lower than in the control groups. As described in the Methods section, this study’s population was randomly assembled. We did not adjust our statistical analyses for the significant differences between the BC and control groups because (1) the primary aim of this study is to describe the subjective quality of life in the three groups, and (2) such differences are expected given the structure of military organization and the distribution of risk across military roles—for example, younger enlisted personnel are often exposed to the greatest combat risk. The current findings are largely in line with our observations in the 5-year follow-up. This emphasises the long-term impact of battlefield injuries at physical and psychological levels and wellbeing and underlines the necessity of long-term care.

The WP subscale of the PDRS, describing the positive impact of work factors experienced by the respondent, was significantly different between the three subgroups in contrast to the non-significant difference found in our 5Y follow-up. The mean and standard deviations of the three groups (BC: 3.732 (0.582), CG1: 3.669 (0.719), CG2: 3.378 (0.742)) how only minor differences and therefore do not suggest a clinically significant difference. In our 5-year follow-up study, 90% of the BC group was still on active duty, compared to 30% in this study. An analogous reduction is observed in the control groups.

No significant differences were found between the groups for the EQ-6D score and the GH subscale of the SF36. Conversely, both were significantly lower for the BC group during our 5Y follow-up. This observation raises multiple hypotheses, such as a potential decline in the impact of certain injuries after a longer follow-up period. If supported by further evidence, aftercare guidelines could be adjusted to accommodate these findings. However, further and more detailed analysis, including a case-by-case analysis over multiple points in time, is required to elaborate.

These results are also in line with earlier reports on long term impact of physical and psychological symptoms in military personnel [12–14].

## Strengths and limitations

In our survey participants were instructed to select one or more mechanisms of injury (MOI) from a predefined drop-down list. Additional details regarding the MOI were only captured if voluntarily provided in the free-text field. Consequently, the exact mechanism of blast-related injuries, e.g., whether the injury was caused by pressure waves or penetrating fragments, was not determined. Furthermore, the presence or absence of traumatic brain injury resulting from the injury was not specifically recorded in the dataset, which could influence long-term symptoms. The observational character of this study has several limitations. Firstly, the associations between study variables and outcomes that have been determined should not be interpreted as a causality. Secondly, response bias through differing response rates per group cannot be overlooked. To minimize this risk, we standardized the number of reminders sent to each potential participant. Thirdly, ranges and cut-off values from the questionnaires can result in over- or under estimation of the impact described, since these tools cannot encompass the entire quality of life perceived.

Lastly, like our 5Y follow up study, this is a single time point retrospective study, and the multiple point analysis between this study and the former will be a subject for our future research, some of which can be conducted using data gathered in this study. We did not take the current occupation and the amount of physical and/or psychological therapy received by the individuals in this study into account in our analysis. A strongpoint of our study is that we have compared the group of injured servicemembers to a group of colleagues who served during the same timeframe and in the same theatre of war for the same nation.

### Future work

The DNBI group was not included in our analysis, because we wanted to focus on the impact of battlefield casualties. Further research could be performed comparing the long-term impact of non-battlefield injuries and battlefield trauma as well as the influence of coping mechanisms and other personal factors [15,16].

In this and our previous follow-up study we have focused on the results as a whole. In future research we wish to perform a longitudinal analysis, analysing the development of stress levels and QOL over time. For future research, exploration of the association between demographics and personal characteristics and interactions with questionnaires could be of added values

### Recommendations

Based on these results of the ten-year follow-up study, the following recommendations can be made: (1) periodical evaluation: It is suggested to implement regular, ongoing assessments of the physical and psychological health of battle casualties (BCs). A systematic, periodic evaluation every 3–5 years can help track the progression of both trauma-related distress and physical health decline over time, ensuring that interventions are timely and relevant; (2) comprehensive assessment of care needs: A tailored approach to care should be prioritized, accounting for both physical and mental health factors. Regular assessments should address the evolving needs of the BC group and adapt treatment plans accordingly, ensuring that any changes in distress levels or physical functioning are adequately managed; (3) balancing disease burden and health: it is suggested to strike a balance between the burden of disease and the individual’s overall health. Focusing on improving quality of life (QOL) while managing the distress related to both physical injuries and psychological trauma may help service members achieve a better reintegration into society and/or the workforce. This approach should also guide future rehabilitation strategies.

## Conclusion

In conclusion, we have found that after 10–15 years, servicemembers who have sustained a battlefield injury still experience significantly more psychological stress and a lower QOL than non-injured servicemembers. This is predominantly on the domain of posttraumatic stress, represented by intrusion, avoidance and hyper arousal, in addition to a reduced (perception) of physical functioning. The findings of this study accentuate our obligation to provide long term care for those who we put in harm’s way. We have provided three recommendations to improve the quality of aftercare based on our observations (1) periodical evaluation every 3–5 years, (2) comprehensive assessment of care needs and (3) balancing disease burden and health to optimize societal and/or workplace integration.
