# Correction: Long-term impact of battle injuries; Ten-year follow-up of Dutch servicemembers injured in Afghanistan

**DOI:** 10.1371/journal.pone.0357223

**Published:** 2026-08-27

**Authors:** Benjamin L. Turner, Thijs T. C. F. van Dongen, Floris Idenburg, Loes de Kruijff, Eelco P. Huizinga, Eric Vermetten, Erik Hoencamp, Rigo Hoencamp

The following information is missing from the Acknowledgment section: Disclaimer: The opinions or assertions contained herein are the private views of the authors and are not to be construed as official or reflecting the views of the Department of Defense or Dutch government. The authors are employees of the Dutch government.
